# Milestone to Ensure Safety and Efficacy of Companion Diagnostic (CDx) That Support Treatment Decisions in Cancer Patients

**DOI:** 10.3390/diagnostics16010155

**Published:** 2026-01-04

**Authors:** Sulim Kang, Sungmin Kim

**Affiliations:** Department of Regulatory Science for Bio-Health Medical Device, Dongguk University-Seoul, 26, Pil-dong 3-ga, Jung-gu, Seoul 04620, Republic of Korea; slkang2047@gmail.com

**Keywords:** companion diagnostic, checklist, safety, efficacy, performance

## Abstract

As demand for biomarker-based companion diagnostics (CDx) tests in clinical oncology of precision medicine increases, a clear understanding of the regulatory framework (especially analytical and clinical performance) is imperative to ensure the safety and efficacy of CDx in enhancing patient quality of life and aiding in treatment decisions. This study analyzes the regulatory policies and approval reports in major countries and identifies regulatory checklists for the pre- and post-marketing analytical and clinical performance to ensure safety and efficacy of CDx. It categorizes the pre-marketing analysis into four commonly used techniques, IHC, FISH, PCR, and NGS, reflecting the diversity of CDx types. All analyses are grounded in the latest regulations and guidelines. The developed checklists were subjected to feasibility assessment by industry experts. Our analysis revealed that there are differences in the pre- and post-marketing regulatory frameworks for CDx, reflecting unique characteristics of each country. In particular, differences were observed in the safety and efficacy assessment methods applied to the platform based on technological principle. Evidence-based checklists are established, which support manufacturers in implementing efficient practices and creating systematic regulatory strategies. Furthermore, these checklists facilitate global market access, activate R&D, enhance clinical implementation, and improve licensing practices.

## 1. Introduction

Personalized diagnostics represents a paradigm shift from the traditional nonspecific approach of conventional oncology drug development to precision medicine, which employs biomarkers for targeted therapy of cancer [1]. Central to this are the companion diagnostic tests (CDx), that significantly enhance the ability to meet unmet medical needs by enabling targeted drug administration to cancer patients [2]. The accuracy of CDx is pivotal in treatment decisions of cancer. False-positive results can result in unnecessary interventions through inappropriate drug administration, while false-negative results can prevent patients from receiving needed treatments despite disease progression. Such inaccuracies can pose serious health threats [3]. Various biomarkers and platforms are utilized for CDx testing, including Immunohistochemistry (IHC), Fluorescence in situ hybridization (FISH), and Polymerase Chain Reaction (PCR). Notably, the use of Next-Generation Sequencing (NGS) has significantly increased in recent years [4,5]. Among these, PCR is the most widely employed technology, accounting for 41.5% of its use in clinical trials [6].

Globally, with the increasing demand for CDx, FDA has been developed enhanced regulation and guidelines for CDx, as has the European Commission when it developed the IVDR regulations in Europe [7,8,9,10,11,12,13,14]. Specifically, the transition to IVDR necessitates a comprehensive review by the Notified Body, stringent performance and clinical evidence requirements, and demands extensive clinical evidence, both pre-and post-marketing [8,15]. However, while these strengthened regulations ensure patient safety by maintaining test quality in clinical environments, they also impose significant challenges on manufacturers due to increased costs, complexity, and the requirement to conduct new clinical studies to comply with regulatory demands [16,17,18]. Additionally, the importance of pre- and post-market management is growing, as maintaining consistent device performance through the development process can be challenging due to the complex regulatory framework; this management is crucial for delivering personalized treatments because it ensures long-term safety and efficacy, and helps generate clinical evidence [19,20,21]. Notably, since the FDA and the European Medicines Agency (EMA), enforce strict regulations concerning research progression and the reporting or submission of trial activities, understanding these regulatory requirements is vital for minimizing compliance issues and avoiding severe repercussions for manufacturers during pre- and post-marketing clinical trials [22].

Milestones are key checkpoints that allow you to monitor essential activities for project progress management. By verifying the critical items necessary for a project’s success at each stage or according to set criteria, it aids in managing the overall project schedule to ensure timely completion without delays. milestones are applicable at all key points from the beginning to the end of a project, including project contracts and initiation, interim reports, and final reports. However, since only the critical items are monitored during the project’s progress, it is challenging to identify other elements that may not be crucial but are still necessary for project progress, thus requiring caution during project execution [23]. In Korea, the ‘Medical Device Regulatory Science Milestone’ was released by the Ministry of Food and Drug Safety (MFDS) in July 2022. This milestone is designed to help verify data requirements and readiness for approval preparation, supporting successful product development throughout medical device development. Furthermore, the milestones were updated in 2023, and the ‘Medical Device Regulatory Science Milestone Preparation Guide’ was distributed, providing a guide for utilizing the milestones by incorporating writing tips for each stage of product development, the underlying laws and judgment criteria, and reference information [24].

Previous studies have analyzed various studies on pre- and post-marketing requirements due to the strengthening of CDx regulations, major changes, and potential impacts [15,25,26,27,28,29,30,31]. In studies developing milestones, these were primarily crafted as practical guides to monitor disease progression and advance the expertise and capabilities of medical professionals [32,33,34,35,36,37,38]. However, no literature was found that derived implications and developed milestones through the application of institutional analysis such as guidelines and approval report analysis, focusing on securing safety and efficacy before and after the marketing of CDx as a distinct topic. Therefore, research aimed at developing a guide to support efficient practical performance by manufacturers and clarify regulatory requirements and processes may be beneficial.

In this study, we proposed checklists of a regulatory framework by comparing regulatory requirements by country (US FDA, European EMA, Japanese PMDA, and Korean MFDS) and analyzing approval reports to ensure the pre- and post-marketing safety and efficacy of CDx, evaluating its validity through feedback from a panel of industry experts.

## 2. Methods

### 2.1. Requirements by Regulatory Bodies (US FDA, EU EMA, JP PMDA, KR MFDS)

Guidance documents and legislation for CDx are being implemented worldwide, with their strength increasing [7,8,9,10,11,12,13,14,39,40,41,42,43,44,45,46,47,48,49,50,51]. As of December 2023, major revisions to CDx-related guidelines or legislation are being conducted by six regulatory authorities, as detailed in Supplementary Material Table S1. Furthermore, efforts are underway to establish CDx-specific guidelines in Canada, the UK, and Singapore. The scope of this study encompassed the regulatory environments of four regulatory bodies (FDA, EMA, PMDA, and MFDS), with the selection criteria including: (1) the existence of a dedicated regulatory body for companion diagnostics or other in vitro diagnostic devices, (2) a history of regulatory approvals for companion diagnostics, and (3) at least five years of experience in drafting guidelines following the issuance of the initial companion diagnostics guidance document.

Regulatory guidelines and guidance documents issued by each agency were compiled to examine and analyze regulatory requirements mandated by each regulatory authority. From the collected data, documents specifically pertaining to CDx were selected.

A comparative analysis was performed on the content of drug clinical trials demonstrating analytical performance, clinical performance, clinical efficacy, or usability. These comparative analyses extensively reviewed evaluation scales, key factors, and methods of collecting evidence and indicators. Requirements managing post-marketing safety and efficacy were identified, followed by a comparative analysis across regulatory authorities. This analysis primarily focused on the system’s purpose, data requirements, and methods of data collection.

### 2.2. Analysis of Approval Reports Related to Safety and Efficacy

Generally, we analyzed the approval reports based on both analytical and clinical performance test data that demonstrate the safety and efficacy of in vitro diagnostic medical devices. Particularly, considering the CDx characteristics that demand clinical efficacy or usability data validated through drug clinical trials, we further examined the clinical trial data of drugs where CDx was utilized. The data were segregated based on the four principal technological modalities of CDx (IHC, FISH, PCR, and NGS) and then one set with the most items evaluating analytical performance was chosen. To ensure diversity in devices, products were selected from different countries [52,53,54,55,56,57,58,59,60,61,62,63,64]. From the selected data, we extracted information on the device’s safety and efficacy and analyzed the analysis methods and results according to the evaluation items. In Europe, since CDx has not yet been incorporated into the European Database on Medical Devices (EUDAMED), approval reports were unobtainable and thus excluded from our analysis. Inaccessible data were supplemented with data from national studies or recommendations from multilateral working groups. Furthermore, we explored the data and formats required for submission alongside post-marketing studies, and where possible, we formatted the collected data to establish a foundation for preparing the checklist documentation.

### 2.3. Technical Classification of CDx

CDx development varies depending on the technology employed, and in this study, we examined four technologies (IHC, FISH, PCR, and NGS). IHC involves the use of specific antibodies to detect certain antigens or proteins in tissues, cultures, or smeared cells [65]. FISH employs a nucleic acid probe to hybridize the nuclear DNA of interphase cells or metaphase chromosomes affixed to a microscope slide [66]. PCR is used to amplify specific DNA fragments through a simple enzymatic reaction [67]. NGS is an advanced genetic sequence analysis technology that rapidly deciphers base sequences on a large scale from small DNA fragments. This method involves breaking down a single genome into multiple fragments, reading each fragment simultaneously, and then reconstructing the sequence using computer technology [68].

### 2.4. Validity Assessment

The validity assessment was conducted twice: initially at the study’s onset and subsequently after deriving the final results. Initially, four experts with over ten years of experience in the CDx or in vitro diagnostic medical device field evaluated the study’s necessity, purpose, method, and expected outcomes. The second assessment took place post-development of the final results, termed checklists, with the checklist’s validity verified by 20 industry experts.

#### 2.4.1. In-Depth Interviews with Experts

An in-depth interview was conducted before deriving the final results with four experts in the Korean CDx industry to validate the necessity, direction, methodology, and findings of this study. The interview questionnaire can be found in Supplementary Material Table S2.

#### 2.4.2. Advisory Committee Survey and Opinion Collection

From 8 April to 25 June 2024, a written survey was conducted after deriving the final results with 20 industry professionals engaged in CDx-related fields in Korea, or those preparing for CDx development and commercialization. This survey aimed to verify the applicability of the checklists established in this study on a 5-point scale. The survey questionnaire is available in Supplementary Material Table S3.

## 3. Results

### 3.1. Checklist

Checklists consist of pre-market and post-market regulatory frameworks. Pre-market refers to all stages of medical device development prior to regulatory approval and product launch, while post-market refers to regulatory requirements following regulatory approval. The pre-market checklist is organized according to regulatory authority and technical principle, presenting evaluation items by categorizing them into common and technology-specific items. Post-market checklist is structured based on regulatory authority and system. Evaluation items for the checklist were derived by integrating guidelines, guidance documents, and approval materials from various regulatory agencies. The checklist analyzes CDx-related guidelines issued by each regulatory agency and actual approval reports to define specific performance items. Furthermore, it differentiates between common regulatory requirements and technology-specific requirements. It is segmented into pre-market and post-market phases, as illustrated in Table 1 and Table 2.

### 3.2. Validity Assessment

#### 3.2.1. In-Depth Interviews with Experts

The participants’ fields of work were diverse, encompassing research and development, licensing, and clinical sectors, and they primarily dealt with CDx and other in vitro diagnostic medical devices. All had over 10 years of experience, and among them, three, excluding one, confirmed they had clinical experience. Further details are available in Supplementary Material Table S4.

The major challenges faced by the Korean industry concerning the clinical regulation of CDx include the difficulty in formulating clinical strategies arising from device diversity and the absence of clear guidelines, procedures, or manuals. The primary solution identified involves developing such guidelines or creating an efficient clinical design process.

The overall research outline underscores the importance and necessity of research on CDx, vital not only for enhancing the market and medical economy but also for improving patient welfare. Although it remains underdeveloped, it is timely to undertake research that considers each country’s specific circumstances. It suggested the need for preliminary research into market status and a survey on the status of global guidelines combined with an analysis of related research trends. The majority opinion supported the validity and purpose of the research, concluding that research facilitating efficient practical implementation from the manufacturers’ perspective is essential.

To effectively identify and address the limitations and challenges of CDx, it is crucial to actively analyze and utilize trends and approval reports from advanced countries like the US FDA. Therefore, it was proposed that research incorporating a comprehensive analysis of both domestic and international approval reports, including details of pharmaceutical clinical trials, is necessary. This research should potentially be validated through expert opinions or interviews that reflect real-world insights.

From the company perspective, establishing clear methodologies and guidelines for implementing CDx procedures and processes was deemed beneficial for many firms. Additionally, it is anticipated that such clarity will foster policy adaptation to stimulate regulatory improvements and the development of Korean-specific technologies. The findings of this study are expected to be extensively utilized in the future to enhance interest and applications in relevant industries and fields.

#### 3.2.2. Advisory Committee Survey and Opinion Collection

The majority of the participants (20 individuals in total) worked in the licensing field (41%), dealing primarily with CDx and in vitro diagnostic medical devices. Ninety percent had over five years of work experience, and more than 85% had clinical experience or were currently involved or preparing for it. Detailed information is provided in Supplementary Material Table S5.

We analyzed the respondents’ evaluation opinions on the research background, purpose, methodology, and primary results of this study. The analysis of the 5-point scale evaluation for each element revealed that all items received an average score of 4 or higher, with detailed information available in Supplementary Material Table S6.

In addition, we collected subjective opinions on the overall flow of this study, which encompasses the research background, purpose, and method, as well as the main results. Detailed information is provided in Supplementary Material Table S7. The main comments highlighted that this study offers a systematic approach to CDx development, emphasizing the significance of guidance that addresses various aspects of the entire process, including pre- and post-marketing safety and efficacy verification. This is crucial to minimize trial and error, thereby easing the development process. Moreover, similar to the in vitro diagnostic medical device checklist commentary, which is effectively utilized in the field for new product development, the findings from this study are also deemed valuable. They provide a checklist to assess requirements at each stage, both before and after marketing. Since the required performance varies depending on the usage purpose and technical principle, it was recommended that a detailed study be conducted. This would involve policy research applicable to all CDx variations and some extent of follow-on CDx approval, enabling companies to strategize and leverage their product development efforts. Additionally, given that CDx technology must ensure efficacy and safety verification of drugs unless it involves indication expansions for approved drugs (application to other cancer types) or technology compatibility verification for the same target, a checklist that encompasses both drugs and medical devices should be developed from a regulatory perspective. The results of this study are suggested to substantially aid in the development of CDx.

## 4. Discussion

In this study, we developed regulatory framework checklists by comparing regulatory requirements across regulatory bodies (FDA, EMA, PMDA, and MFDS) and analyzing approval reports to ensure pre- and post-marketing safety and efficacy of CDx. As the demand for CDx has grown due to a paradigm shift in oncology, and as its clinical importance has escalated, clinical regulations worldwide are being strengthened [69,70]. This poses an additional challenge for manufacturers in the clinical implementation of CDx, necessitating a clear understanding of pre- and post-marketing safety and efficacy regulations [71]. We aimed to develop a checklist that addresses a gap identified in previous research: a lack of literature that draws conclusions from analysis of approval reports based on differences in regulatory requirements across regulatory agencies. These checklists are intended to enhance the clinical implementation supported by R&D personnel and industry staff, including CDx manufacturers (importers).

In the pre-marketing regulatory framework analysis, we investigated and compared regulatory requirements for safety and efficacy by regulatory authority, analyzing approval reports based on technology principles to identify detailed evaluation items or methods. Regulatory authorities commonly require that safety and efficacy ensure accuracy and reliability depending on the intended use. Drugs’ clinical trial data may be necessary to secure clinical efficacy or usability, considering the nature of CDx, which can be corroborated by analytical and clinical performance. However, unique characteristics arise from differences in safety and efficacy verification requirements by regulatory authorities [72,73,74].

The FDA underscores the link between the output of the device and its intended use, aligned with clinical reference standards. This context mirrors the requirement for method comparison studies with existing products or technologies per guidance documents, suggesting consistency with studies anticipated to be conducted based on standardized performance studies like those of the Clinical & Laboratory Standards Institute [26,75,76]. To prove analytical performance, samples matching the pathological characteristics of appropriate patients should be collected and preserved, and clinical performance should entail collecting clinical trial data on drugs, which includes the ability to predict cancer patient treatment outcomes. Additionally, if the device slated for final commercialization is unusable in research, prototype testing or bridging studies demonstrating similarity to clinical trial assays may be conducted [26,77,78].

The EMA aims for scientifically proven safety and intended clinical benefits of the device. Because the efficacy of a medicinal product closely correlates with the device’s performance, the benefit-risk balance of the device’s accuracy and specificity must be thoroughly assessed, given that treatment decisions are significantly influenced by this high correlation [27,79]. EMA guidance dictates that the studies vary depending on the anticipated development scenario, supported by scientific validation, analytical performance, and clinical performance data [30,80]. After scientific validation assessing whether the biomarker can signify physiological status or clinical importance [81], analytical performance should be demonstrated by evaluating the biomarker detection rate in the device [18,82]. Clinical performance is assessed based on the correlation between cancer patient eligibility and pathophysiological status; the studies performed may vary depending on whether the device is being developed in conjunction with a medicinal product.

The Pharmaceuticals and Medical Device Agency (PMDA) evaluates the intended use and its impact on safety and efficacy based on clinical significance [83]. It is crucial to determine whether the device’s detection capability remains consistent before and after a change in the intended condition. If the device is not used in a confirmatory clinical trial of a drug, its consistency under the identical conditions of the clinical trial should be assessed. Generally, clinical performance should be validated through a prospective randomized controlled trial in a confirmatory clinical setting, although retrospective studies may also be feasible in some cases. The PMDA guidelines emphasize the inclusion of biomarker-negative patients in the early stages of clinical trials, contrasting with other regulatory bodies. This approach indicates that potential safety and efficacy should be gauged through analyzing the risk-benefit balance between biomarker-positive and -negative patients [84].

The MFDS evaluates the device based on its intended use, the rationality of safety and efficacy, and the risk level [85]. The guidelines stress that since the device’s performance directly affects the safety and efficacy of the treatment prescribed, a risk factor-based approach should be used to assess the risk levels associated with false positives and false negatives [13,86]. As a research method, compatibility with existing test methods must be demonstrated. Drug reactivity using CDx should be confirmed, and equivalence with existing products may be assessed.

License data analysis confirmed key evaluation items and analytic methods according to the technology principle and guideline-required evaluation indicators. Since the technology principle influences the device’s operating principle, characteristic items are discernible in the performance analysis, which can be verified by dividing it into preprocessing and analysis stages according to technology. NGS was characterized by comparing it with PCR, guard banding at each workflow stage (sample eligibility, library preparation, sequencing) [87,88], and PCR required details on DNA input, minimum tumor content, and the specificity of primers and probes [89]. IHC and FISH necessitated performance evaluations in terms of staining intensity and the time required for the analysis procedure. It was determined that IHC needed an analysis of antibody specificity and reagent compatibility, while FISH required analysis concerning probe concentration and photostability. Notably, the FDA was found to perform the most comprehensive evaluations in analyzing performance. This indicates that a substantial amount of analytical data may be required, as the FDA expects most companies outside the United States to conduct studies at least three different sites [74]. In terms of clinical performance, when using clinical trial data for a drug, it is imperative to confirm improvements in progression-free survival, objective response rate, or overall survival [90,91,92]. Additionally, when comparing with or assessing equivalence to existing methods, measures such as overall percent agreement, positive percent agreement, and negative percent agreement must be evaluated [93,94]. Consequently, the studies required to demonstrate the safety and efficacy of a device may differ based on the technology and platform used in the device, as well as its intended use [26].

The analysis of the post-market regulatory framework included a comparative examination of safety and efficacy regulatory requirements by various authorities. Consequently, post-market study data from public databases were analyzed, and the submission materials and formats were assessed. It was confirmed that differences exist in the post-market systems of each country regarding the regulatory requirements for safety and efficacy. The FDA’s Post-Approval Studies Program (PAS) mandates the collection of data in a post-market setting as opposed to pre-marketing when the specific effects or risks associated with a device remain uncertain and requires the submission of both interim and final reports [95,96]. The EMA stipulates the detailed collection of market experience post-marketing. Specifically, the Post-Market Performance Follow-up (PMPF) mandates the reporting of risks or performance issues not known before marketing and necessitates regular performance updates through the Periodic Safety Update Report (PSUR) [97]. The PMDA requires data collection through use history surveys, post-market database investigations, and post-market clinical trials. Moreover, if designated by the Ministry of Health, Labour and Welfare, a reevaluation may be undertaken requiring the submission of information gathered in an actual clinical setting [98]. Additionally, the construction of an infrastructure for a post-marketing database is underway, hence securing Real-world Evidence/Real-world data may be essential. The MFDS demands clinical performance tests and technical documentation as evidence under the reevaluation system that gathers new information concerning the safety and efficacy of approved devices [99]. Furthermore, a renewal system is in place that mandates review every five years for efficient management.

Analysis of the approval reports enabled confirmation of the data items and submission materials for each regulatory authority’s system. The PAS necessitates a study design including objectives, population, evaluation variables, follow-up devices, or evaluation frequency, and the PMPF must incorporate performance related to equivalent or similar devices, latest technology, relevant standards, and guidelines. The PSUR must contain a risk-benefit analysis, key findings, sales volume, and frequency of use of the device, while the PMDA’s Post-Marketing Safety must detail the subjects, investigation period and items, and the analysis method for each item. The reevaluation process of the MFDS can incorporate academic papers, clinical trial data, product descriptions, and government agency announcements for safety information, and can include storage methods, use periods or expiration dates, analytical performance tests, quality control tests, standard materials, and specimen storage data as updated data. The checklists consist of checklists developed from an analysis of pre-market and post-market regulatory frameworks. The pre-market checklist is organized according to regulatory authority and technical principle, presenting evaluation items by categorizing them into common and technology-specific items. The post-market checklist is structured based on regulatory authority and system. However, as these checklists are proposed based on critical aspects of CDx safety and efficacy, reliance solely on the presented checklist is challenging, suggesting that familiarization during practical implementation is necessary. Consequently, this checklist serves to compile and verify essential evaluation items and data when preparing for early-stage clinical performance tests in CDx development and subsequent clinical performance tests post-marketing.

The validity assessment was conducted twice: initially at the study’s onset and subsequently after deriving the final results. Initially, four experts with over ten years of experience in the CDx or in vitro diagnostic medical device field evaluated the study’s necessity, purpose, method, and expected outcomes. The second assessment took place post-development of the final results and termed checklists, with the checklist’s validity verified by 20 industry experts. Opinions were gathered on the ‘rearrangement that allows for systematic inspection’ proposed during the validity assessment. Consequently, the items were reordered to facilitate the recording of fundamental data by item and to assess their incorporation, enhancing the feasibility of practical application. This study stands out as it uniquely derived insights by applying an analysis of approval reports, considering institutional procedures for ensuring safety and efficacy of CDx both pre- and post-marketing as an autonomous subject. Moreover, checklists were tailored to each country’s regulatory frameworks, aiming to streamline the challenges manufacturers may encounter into an efficient process. Conversely, the analysis could not incorporate European authorization data as EUDAMED does not apply, resulting in its exclusion. Furthermore, due to transparency issues in information disclosure, inaccessible data was supplemented by studies from the respective countries or recommended data from a multi-stakeholder working group, introducing a limitation as analysis based on actual data was unfeasible. Additionally, because the sample size of the survey is small, generalizations may be limited. The data extraction was confined to activities related to the safety, efficacy, or performance of the device before and after marketing, which may obscure the overall development process comprehension. Despite these limitations, the research scope was deliberately narrowed to ensure consistency, though future studies using actual data could address these gaps.

## 5. Conclusions

In this study, we proposed checklists for the regulatory framework by comparing the regulatory requirements from various countries (FDA, EMA, PMDA, and MFDS) and analyzing approval reports to ensure the safety and efficacy of CDx both before and after marketing. Through this analysis, we confirmed that differences exist in the regulatory frameworks of CDx before and after marketing, reflecting the unique characteristics of each country. Notably, differences were observed in the safety and efficacy evaluation methods applied to the platform based on the technological principle. The identified checklists are anticipated to enhance understanding of the institutional facets that govern the entry of CDx into the global market, as well as the evaluation methods applicable according to the technological principle. This should facilitate the promotion of R&D, clinical implementation, and licensing activities. Consequently, by providing CDx manufacturers and developers with these checklists, they can establish a systematic regulatory strategy and implement efficient practices.

## Figures and Tables

**Table 1:** Pre-market checklists.

Regulatory Authorities	Classification	Checklist
FDA	Commonness	◆ Analytical performance considerations □ Sensitivity □ Specificity □ Accuracy □ Reproducibility □ Precision □ Stability
		◆ Study design □ Prospective □ Retrospective ◆ Clinical performance study method □ Drug clinical trial data collection □ Bridging study □ Comparison of consistency with existing products ◆ Clinical performance considerations □ Study design □ Inclusion and exclusion criteria □ Clinical endpoints □ Population characteristics □ Sampling criteria □ Sample size
	NGS	□ Limit of blank (LoB) □ Limit of detection (LoD) □ DNA Input □ Interference (endogenous and exogenous) □ Equivalence □ PCR system comparison □ Guard banding
	PCR	□ Limit of blank (LoB) □ Limit of detection (LoD) □ DNA Input □ Minimum tumor content □ Number of curls □ Interference (endogenous and exogenous) □ Cross-reaction (specificity of primers and probes) □ Equivalence □ Cross-contamination □ Guard banding
	IHC	□ Antibody specificity □ Pre-analytical variables (slide stability, tissue fixation, etc.) □ Repeatability □ Compatibility between reagent lots □ Robustness of staining procedure
	FISH	□ Normal cutoff □ Robustness □ Probe concentration optimization and limitations □ Microbial interference
EMA	Analytical performance	□ Analytical sensitivity □ Analytical specificity □ Precision □ Repeatability □ Reproducibility □ Accuracy □ Detection and quantitation limits □ Interference response
	Clinical performance	◆ Study design □ Prospective □ Retrospective ◆ Clinical performance study □ Therapeutic clinical trial □ Bridging study □ Concordance study ◆ Clinical performance considerations □ Progression-free survival □ Objective response rate □ Overall survival □ Hazard ratio □ CTA concordance rate (OPA, PPA, NPA)
PMDA	Commonness	◆ Analytical performance considerations □ Accuracy □ Precision □ Reproducibility □ Repeatability □ Cross-reactivity □ Detection limits □ Stability
		◆ Study design □ Prospective □ Retrospective ◆ Clinical performance study □ Clinical trial of therapeutic agent □ Bridging study □ Concordance study ◆ Clinical performance considerations □ Progression-free survival □ Objective response rate □ Overall survival □ Hazard ratio □ CTA concordance rate □ Concordance rate with existing products
	NGS	□ Analytical cutoff □ Comparison with prototype □ Appropriateness of proposed target gene □ Appropriateness of sensitivity for target mutation detection □ Appropriateness of report writing and content
	PCR	□ Control Ct value □ Correlation □ Interference reaction
	IHC	□ Control Ct value □ Correlation □ Fixation, slide sample time and temperature □ Staining protocol □ Interference reaction
	FISH	□ Analytical sensitivity □ Analytical specificity □ Nonspecific reaction □ Probe signal intensity □ Slide background □ Microbiological test
MFDS	Commonness	◆ Analytical performance considerations □ Accuracy □ Precision □ Reproducibility □ Repeatability □ Specificity □ Detection limit □ Analytical cutoff □ Interference response □ Stability
		◆ Study design □ Prospective □ Retrospective ◆ Clinical performance study □ Collection of clinical trial data for therapeutic agents □ Bridging study □ Equivalence assessment ◆ Clinical performance considerations □ Progression-free survival □ Objective response rate □ Overall survival □ Hazard ratio □ CTA concordance rate (OPA, PPA, NPA) □ Equivalence to existing products
	NGS	□ Blank limits □ Tissue content □ Guard banding □ DNA/RNA dose □ Cross-reactivity □ Cross-contamination □ Tissue fixation time □ Interference reactions □ Tumor cell content
	PCR	□ Blank limits □ Gene frequency and content □ Microbiological testing □ Influence of input DNA □ Cross-contamination □ Sample processing variability □ Lot interchangeability
	IHC	□ Analytical sensitivity □ Analytical specificity □ Normal and tumor tissue levels □ Staining background and intensity levels □ Cross-reactivity
	FISH	□ Hybridization efficiency □ Analytical sensitivity □ Analytical specificity □ Cross-reactivity

**Table 2:** Post-market checklists.

Regulatory Authorities	Policy	Checklist
FDA	PAS	◆ Whether applicable □ Review whether PAS applicable
		◆ Plan □ Study design method □ Study purpose □ Study subjects (including negative patients) □ Sample size □ Evaluation variables (1st/2nd) □ Expected follow-up period □ Evaluation method
		◆ Results Analysis □ Study Design □ Study Purpose □ Study Subjects (including negative patients) □ Sample Size □ Evaluation Variables (1st/2nd) □ Follow-up Period □ Results Data
		◆ Interim Report □ Approval Study Report □ Registration Status Report □ PAS Progress Report
		◆ Final Report □ Review of compliance with protocol methodology □ Reasons for deviation from standard □ Results of performance and safety/validity evaluation
EMA	PMS	◆ Plan □ Systematic process for data collection □ Risk-benefit analysis and indicators □ Complaint investigation and field data collection □ Corrective action procedures □ Method of communication with users □ PMPF plan
		◆ Purpose □ Risk-benefit decision analysis □ Design and manufacturing information □ Performance and safety summary updates □ Corrective and preventive actions □ Field safety corrective actions □ Usability and performance/safety improvements □ Follow-up monitoring and reporting
		◆ Data □ Information on serious accidents □ Non-serious or undesirable side effects □ Follow-up management information □ Opinions or literature from relevant experts □ Information provided by users/distributors □ Public information on similar devices
	PMPF	◆ Plan □ Protocols and procedures □ Risk management □ Specific objectives □ Performance of equivalent or similar devices □ Latest technology and relevant performance data □ Relevant CS and standards/guidelines □ Overall schedule
		◆ Methodology □ Confirm safety/performance over the expected life cycle □ Previously unknown risks □ Identification of performance limitations and contraindications □ Identification and analysis of risks based on factual evidence □ Relationship of clinical evidence to risk-benefit ratio □ Identification of potential misuse of the device
	PSUR	□ Risk Benefit Analysis Conclusion □ Key Findings □ Device Sales Volume □ Usage Scale □ Frequency of Usage
PMDA	PMS	◆ Plan □ Operation manual or procedure
		◆ Usage Performance Survey □ Survey Purpose □ Target Scope and Number □ Survey Method □ Survey Period □ Survey Items □ Analysis Items and Methods
		◆ Post-marketing database survey □ Survey purpose □ Description of database used □ Scope and number of subjects □ Survey method □ Survey period □ Survey items □ Analysis items and methods
		◆ Post-marketing clinical trials □ Purpose of the study □ Data on performance □ Data related to the intended use □ Data on effectiveness
	Reevaluation	□ Determination of applicability □ Performance data based on actual clinical environment
MFDS	Reevaluation	◆ Safety Information □ Domestic and international academic papers □ Clinical trial test data □ Product descriptions from foreign manufacturers □ Domestic and international government agency announcements □ Risk management analysis reports
		◆ Data on clinical performance tests □ Clinical sensitivity □ Clinical specificity □ Clinical performance according to product characteristics (when clinical sensitivity/specificity measurement is difficult)
	Renewal	◆ Submission materials □ Original license □ Proof of safety and efficacy maintenance □ Production (import) performance data □ Safety information and action data
		◆ Data reflecting the latest standards □ Data on storage method and period of use or expiration date □ Data on analytical performance test □ Data on quality control test □ Data on standard materials and sample storage

## Data Availability

No new data were created or analyzed in this study. Data sharing is not applicable to this article.

## Supplementary Materials

The following supporting information can be downloaded at: https://www.mdpi.com/article/10.3390/diagnostics16010155/s1, Table S1. CDx Guideline Development Status by Regulatory Authority; Table S2. In-depth interview questionnaire; Table S3. Survey Questionnaire; Table S4. Interviewee Information (Unit: respondents); Table S5. Survey participant information (unit: respondents); Table S6. Survey Results (5-point scale); Table S7. Survey Results (Subjective).

## Abbreviations

The following abbreviations are used in this manuscript:

CTA	Clinical Trial Assay
OPA	Overall Percent Agreement
PPA	Positive Percent Agreement
NPA	Negative Percent Agreement
PAS	Post-Approval Studies
PMS	Post-Marketing Surveillance
PMPF	Post-Market Performance Follow-up
PSUR	Periodic Safety Update Report
