# Supplementary material for: Milestone to Ensure Safety and Efficacy of Companion Diagnostic (CDx) That Support Treatment Decisions in Cancer Patients
Source: Diagnostics (Basel). 2026 Jan 4;16(1):155. doi: 10.3390/diagnostics16010155 (PMC12785362; doi:10.3390/diagnostics16010155)
Supplement: Supplementary file 1 [file diagnostics-16-00155-s001.zip › diagnostics-3971545-supplementary.pdf]

Table S1. CDx Guideline Development Status by Regulatory Authority

Division		Number of approvals	CDx Guideline Development Status
US	US Food and Drug Administration (FDA)	57	1) In Vitro Companion Diagnostic Devices[2014.08]<br>2) Principles for Codevelopment of and In Vitro companion Diagnostic Device with a Therapeutic Product[2016.07]<br>3) Developing and Labeling In Vitro Companion Diagnostic Devices for a Specific Group of Oncology Therapeutic Products[2020.04]<br>4) U.S. Food & Drug Administration. Procedures for Handling Post-Approval Studies Imposed by Premarket Approval Application Order[2022.10]<br>5) Oncology Drug Products Used with Certain In Vitro Diagnostic Tests: Pilot Program[2023.06]
EU	European Medicines Agency (EMA)	-	REGULATION (EU) 2017/746 OF THE EUROPEAN PARLIAMENT AND OF THE COUNCIL of 5 April 2017 on in vitro diagnostic medical devices and repealing Directive 98/79/EC and Commission Decision 2010/227/EU[2017.5]
AUS	Therapeutic Goods Administration (TGA)	≥17	IVD companion diagnostics Guidance on regulatory requirements[2022.10]
JP	Pharmaceutical and Medical Devices Agency (PMDA)	43	1) Notification on Approval Application for In Vitro Companion Diagnostics and Corresponding Therapeutic Products[2013.07]<br>2) Technical Guidance on Development of In Vitro Companion Diagnostics and Corresponding Therapeutic Products[2013.12]<br>3) Notification on Handling of In Vitro Diagnostics and Medical Device Products Aiming for Drug-agnostic Companion Diagnostics[2022.03]<br>4) Guidance on Drug-Agnostic Companion Diagnostics [2022.07]
CN	National Medical Products Administration (NMPA)	-	1) Revision for instructions for use and technical review of tumor companion diagnostics based on similar therapeutic drugs(Draft)[2020.07]<br>2) Technical Review of CDx for the Same Kind of Therapeutic Drug[2020.07]<br>3) Guidance for Clinical Study of CDx for the Marketed Oncology Drugs[2020.08]<br>4) Guidelines for Technical Review and IFU Updates of Tumor Companion Diagnostics Reagents based on Similar Treatment Drugs[2021.04]<br>5) Guidelines for Registration Review of Original Companion Diagnostics Reagents Co-developed (Simultaneously) with Anti-Tumor Drugs[2022.06]
KR	Ministry of Food and Drug Safety (MFDS)	30	1) Guidelines for approval and review of in vitro companion diagnostic devices (Guide for complainants) [2015.10]<br>2) Guidelines for safety, performance, and clinical trial plan evaluation of companion diagnostic medical devices using liquid biopsy (Guide for complainants) [2018.02]<br>3) Explanation of significance verification of NGS-based genetic testing (Guide for complainants) [2022.10]<br>4) Guidelines for approval and review of companion diagnostic medical devices (Guide for complainants) [2022.12]

Table S2. In-depth interview questionnaire

**Participant Pre-Question**	1) What is your field of affiliation?<br>① School/Educational institution<br>② Hospital<br>③ Research institute<br>④ Industry<br>⑤ Other ( )<br><br>2) What is your field of work? (Multiple responses are possible)<br>① Research/Development<br>② Talent training<br>③ Quality control<br>④ Clinical<br>⑤ Other ( )<br><br>3) What is your work experience?<br>① Less than 1 year<br>② 1-2 years<br>③ 3-5 years<br>④ 5-10 years<br>⑤ More than 10 years (years)<br><br>4) Do you have experience in clinical performance testing of in vitro diagnostic medical devices?<br>① Experienced<br>② In progress<br>③ In preparation<br>④ No experience<br>⑤ Other ( )
**Regulatory Issues Relevant Questions**	1. What do you think is the problem facing the industry in relation to clinical regulations for domestic companion diagnostic medical devices?<br>2. What do you think should be the most important solution to your answer to question 1?<br>3. What do you think is the most problematic or important point when collecting clinical evidence for companion diagnostic medical devices?
**Justification for the Need for Research**	1. Is the presented research background appropriate for the overall research flow?<br>2. Are the limitations and supplementary points identified in the previous research survey valid for the research necessity?<br>3. Are the presented research necessity and purpose valid?
**Appropriateness of Research Methodology and Results**	1. Is the entire process of the presented research method appropriate?<br>2. Are the analysis results and detailed analysis methods for the presented research method appropriate?<br>3. Are the research results and conclusions derived through the presented research method appropriate?
**Utilization of This Research**	Please give me your opinion on the usability of the results derived through this research.
**Other**	Please feel free to tell me if you have any other opinions regarding this research.

Table S3. Survey Questionnaire

**Participant Pre-Question**	1) What is your field of affiliation?<br>① School/educational institution<br>② Hospital<br>③ Research institute<br>④ Industry<br>⑤ Other ( )<br>2) What is your field of work? (Multiple responses are possible)<br>① Research/development<br>② Talent training<br>③ Quality control<br>④ Clinical<br>⑤ Other ( )<br>3) What is your work experience?<br>① Less than 1 year<br>② 1-2 years<br>③ 3-5 years<br>④ 5-10 years<br>⑤ More than 10 years (years)<br>4) Do you have experience in clinical performance testing of in vitro diagnostic medical devices?<br>① Experienced<br>② In progress<br>③ In preparation<br>④ No experience<br>⑤ Other ( )
**Validity of research results (5-point scale)**	1. Among the milestones derived from the results, do you think the 'Pre-market Clinical Validation Checklist' is helpful in carrying out your work?<br>2. Among the milestones derived from the results, do you think the 'Post-market Clinical Validation Checklist' is helpful in carrying out your work?
**Other**	If you have any other opinions about this study, please feel free to write them down.

Table S4. Interviewee Information (Unit: respondents)

Division	Answer				
Field of work	R&D	Licensing	Quality Control	Clinical	Other
	1	2	0	2	2
Product line	CDx	IVD (Excluding CDx)	Medical device	Other	
	2	2	0	0	
Work experience	less than 1 year	1-2 years	3-5 years	5-10 years	More than 10 years
	0	0	0	0	4
IVD clinical experience	Experienced	In progress	In preparation	No experience	Other
	3	1	0	1	0

Table S5. Survey participant information (unit: respondents)

Division	Answer				
Field of work	R&D	Licensing	Quality Control	Clinical	Other
	5	11	2	6	3
Product line	CDx	IVD (Excluding CDx)	Medical device	Other	
	10	14	6	3	
Work experience	less than 1 year	1-2 years	3-5 years	5-10 years	More than 10 years
	0	1	1	6	12
IVD clinical experience	Experienced	In progress	In preparation	No experience	Other
	15	4	3	3	0

Table S6. Survey Results (5-point scale)

Category	5-point scale response results					
	**1**	**2**	**3**	**4**	**5**	**Average**
**Research background**	0	0	3	9	8	4.25
**Research purpose**	0	0	4	7	9	4.25
**Research method**	0	0	1	12	7	4.3
**Pre-marketing Checklist**	0	1	3	9	7	4.1
**Post-marketing Checklist**	0	1	3	8	8	4.15

Table S7. Survey Results (Subjective)

Category	Detail
**Research background**	• It is both commendable and regrettable that guideline studies are only now emerging, given the long-standing concept of precision medicine.<br>• It would be beneficial to underscore the significant role that advances in biotechnology, genomics, and bioinformatics have played in the development of precision medicine and personalized treatment, and to discuss how these technologies have enabled the creation of companion diagnostics.<br>• I believe the research background presented is highly appropriate for the overall research trajectory. The implementation of precision medicine, the significance of personalized treatment, and the need for biomarker-based companion diagnostic tests are currently pivotal topics in medicine. Moreover, the growth of companion diagnostic medical devices and the consequent surge in market demand further underscore the importance of this research. As such, it will likely lay a solid foundation for practical advancements in companion diagnostics.<br>• Companion diagnostic medical devices offer significant patient benefits, but inaccurate results pose serious risks; thus, precise diagnostic performance is crucial. Additionally, due to the nature of these products, which require concurrent drug review at the approval stage, developers are likely to encounter substantial regulatory challenges during clinical trials or approval processes. Collecting case studies from regulatory bodies and establishing guidelines will be instrumental in swiftly bringing high-performance products to patients.<br>• The advancement of the proposed research project is anticipated to significantly enhance the technological development and market growth of the companion diagnostic medical device sector, as well as strengthen the competitiveness of domestic firms. Ultimately, this is expected to improve patient treatment outcomes and the quality of medical services.
**Research purpose**	• With the rising demand for CDx, the need for companion diagnostic devices featuring enhanced sensitivity, specificity, and accuracy is increasingly urgent. Consequently, a comprehensive understanding of the regulatory requirements of the FDA, IVDR, PMDA, etc., is essential for global progression.<br>• The necessity and purpose of the proposed research are considered very valid. In the current era, international regulations are being strengthened along with the increasing demand for CDx, posing a new phase of challenges for in vitro diagnostic medical device manufacturers. It highlights the importance of maintaining device performance, managing post-marketing activities, and understanding regulatory processes clearly. Against this backdrop, this study appears well-suited to propose a milestone for the clinical regulatory framework by comparing regulatory requirements by country and performing case analyses to ensure safety and efficacy before and after the marketing of companion diagnostic medical devices.<br>• The proposed research project is expected to support patient-tailored treatments, thereby improving the quality of medical care and ensuring the safety and effectiveness of medical devices. The necessity and purpose of this project are reasonable. Moreover, the growth of companion diagnostic medical device technology in the current global market is rapid and attempts to develop technology accordingly are insufficient. Therefore, this study will help to identify the latest trends and promote the development of domestic technology, validating its purpose and necessity.
**Research method**	• This study is useful as it presents a timeline corresponding to the procedural guidelines of domestic and international institutions, serving as a practical reference. The research method is hence deemed reasonable. The methodology is expected to enhance understanding and compliance with regulatory requirements and is positively noted for incorporating feedback from the initial survey.<br>• In post-marketing clinical trials, collecting data from the databases of regulatory agencies might pose challenges.<br>• If a systematic literature review is utilized, it is expected to significantly aid manufacturers

Category	Detail
	in conducting clinical trials by providing an analysis and presentation of the design direction of clinical performance tests of companion diagnostic devices, sample selection, and efficacy indicators.<br>• It is crucial to differentiate between pre-marketing and post-marketing phases, and it is also necessary to analyze the appropriate timing, methods, and collaborations between pharmaceutical companies and developers of companion diagnostic medical devices.<br>• In addition to analyzing actual research performance data from regulatory agencies, it is essential to incorporate a quantitative analysis methodology that numerically assesses the impact of regulatory changes on the development, market entry time, and cost of companion diagnostic devices.
**Pre-marketing Checklist**	• Organizing various guidelines into a table allows for effective comparison and evaluation, enhancing our understanding of each country's review perspectives. Utilizing the checklist in our work appears beneficial.<br>• This reflects the essential elements for evaluating the product's effectiveness and stability accurately.<br>• Using performance evaluation data from pre-approval stages can significantly aid the CDx approval process.<br>• Reviewing the pre-market clinical validation checklist and commentary for each country is beneficial for practical application.<br>• The pre-market clinical validation checklist and commentary are invaluable for developing companion diagnostic medical devices. Systematic approaches such as clinical performance study methods, study design, and inclusion and exclusion criteria are facilitated, and methodologies like bridging studies or consistency comparisons with existing products prove highly useful in application. The presentation of approval cases was particularly informative. However, reliance solely on past approval cases might lead manufacturers to encounter unforeseen issues due to discrepancies with current regulations and guidelines during the actual approval application.<br>• While presenting clinical performance tests and approval cases is advisable, actual products may often deviate from these examples. To enhance practical usability, it would be beneficial to present additional products in question.<br>• Checklists and explanatory notes enable the evaluation of safety and initial effectiveness of medical devices, crucial for delivering safe and effective medical services. We anticipate that this will standardize procedures, enhance quality in the medical field, reduce errors, and manage resources efficiently.<br>• This approach will assist in practical implementation by enabling the verification of application item requirements based on major regulatory authorities and by providing examples of necessary test items.<br>• Providing milestones and explanatory notes in terms of practical response will serve as useful materials. Presenting the process in a flowchart to capture the entire sequence at a glance for each milestone, accompanied by a checklist and commentary, would enhance comprehension.<br>• This research should be presented as a milestone through a more systematic review and analysis of the data, rather than a simple list.<br>• Although useful, the analytical performance of a companion diagnostic device is generally comparable to that of other in vitro diagnostic medical devices. Moreover, companion diagnostic devices, even those based on different principles, require nearly identical performance verification. Therefore, presenting a method for creating a consortium with pharmaceutical companies and medical institutions to strategically evaluate clinical performance would be beneficial.
**Post-marketing Checklist**	• Reviewing the post-marketing clinical verification checklist and commentary for each country is beneficial and aids in practical implementation.<br>• It accurately captures the essential elements necessary for assessing the product's effectiveness and stability.

Category	Detail
	• The post-marketing clinical component can be applied in risk management and adverse event reporting for companion diagnostic medical devices used in monitoring disease prognosis, providing valuable baseline data.<br>• This process evaluates the safety and initial effectiveness of medical devices and is crucial for delivering safe and effective medical services to patients. Standardized procedures are also expected to enhance medical quality, minimize errors, and enable efficient resource management.
